# Brexit and the European National Health Service England Workforce: A Quantitative Analysis of Doctors’ Perceived Professional Impact and Intentions to Leave the United Kingdom

**DOI:** 10.5334/aogh.3048

**Published:** 2021-06-24

**Authors:** Adrienne Milner, Rebecca Nielsen, Ashton M. Verdery

**Affiliations:** 1 Department of Health Sciences, Brunel University London, Mary Seacole Building, Uxbridge UB8 3PH, United Kingdom; 2 Barts and The London School of Medicine and Dentistry, Queen Mary University of London, Yvonne Carter Building, 58 Turner Street, London E1 2AB, United Kingdom; 3 Department of Sociology and Criminology, Pennsylvania State University, 712 Oswald Tower, University Park, PA 16801, United States

## Abstract

**Background:**

Although survey data suggest that Brexit has negatively influenced European doctors’ decisions to remain in the United Kingdom, this is the first quantitative study to use multivariate analysis to explore this relationship.

**Objective:**

To assess how Brexit relates to doctors’ migration intentions in relation to their feelings that Brexit has impacted their professional life, national identity, and demographic factors.

**Method:**

We collected data from 59 self-reported EU/EEA/European identifying doctors working in the UK. We weighted results to the English National Health Service population in terms of gender, professional grade level and ethnicity and ran weighted regression analyses of respondents’ plans (leaving, considering, not considering) and whether they reported Brexit influencing their decision-making. We then examined how stating that Brexit affected their career, national identity, and sex and age related to doctors’ intentions to leave or stay.

**Findings:**

The more doctors agreed that Brexit had impacted their professional lives, the more likely they were to state they intended to leave the UK. We found doctors with increased levels of British identity less likely to leave than those with reduced British identity. Interestingly, we found that those with higher levels of European identity were less likely to leave but more sharply likely to consider leaving compared to those with lower levels of European identity.

**Conclusions:**

Respondents reported large professional impacts of Brexit. To retain these individuals in the British medical system, the NHS should provide financial and legal assistance for those applying for settled status and financial and other incentives comparable to what doctors could receive in EU health systems.

## Introduction

The COVID-19 pandemic highlights not only the importance of healthcare workers, but also that stability of health systems is essential. Although much recent scholarly and public attention has been devoted to how the National Health Service (NHS) can cope with COVID-19, Brexit continues to be a vital factor in determining the future of the NHS. The NHS was a central focus of the ‘Leave’ side of the 2016 Brexit debate, with advertisements promising an extra £350 million per week devoted to the NHS. Yet, the reality is vastly different nearly five years later. The British Medical Association and Nursing and Midwifery Council oppose Brexit as they believe it will be harmful to the NHS [1,2]. It is still unclear what the future relationship between the European Union (EU) and the United Kingdom (UK) will be, however if forecasting is correct, the NHS will feel substantial negative economic repercussions and deregulation will lead to further squeezes on central NHS funding, currently not being kept to adequate levels [3,4]. Previously, due to the political uncertainty and political volatility prior to the December 2019 General Election, the NHS, like many other institutions, has used substantial funds (£2.3 billion) [5] and resources on no-deal planning, which could have been spent instead in resource-deprived areas. Furthermore, Brexit is likely to change the way trade, competition, scientific research, pharmaceuticals and medical equipment are regulated. It is against this background that European (EU and EEA) doctors are choosing whether to stay in this workplace or to leave the UK. Doctors who qualified in the EU or EEA make up 10% of doctors in the NHS [6] and additionally considering others who may also have connections to Europe, potential losses of these staff are significant.

Beyond the domain of healthcare, Brexit has had, and will continue to have, effects both on a personal level and practical level for all of the 3.7 million Europeans in the UK [3,7]. For healthcare professionals specifically, the decision to move to another or stay in a country is a two-tiered decision. For doctors who are choosing a health care system in which to work, there are both push and pull factors impacting their decision related to pay, working hours and support at work. They are also choosing a place to live which may impact their family and as such, factors such as sense of community cost of living and importantly in the context of this paper, their connectedness to the country could potentially influence European doctors’ Brexit plans. This paper focuses specifically on demographics, such as age, gender, career level, marital status, partner nationality and personal identification with their country of origin and the UK of those European doctors to examine how Brexit may relate to their decisions to potentially leave the UK.

### Brexit and the NHS as a workplace

Even prior to COVID-19, the challenging working conditions in the NHS have been widely reported, both in the media and academic literature, and these have been exacerbated by the pandemic. Difficult NHS working environments may partially be attributed to lack of staff, with 11,000 advertised medical vacancies in addition to reported shortages of doctors in 75% of all specialties in 2016 [8]. Shortages in all professional staff are reflected in the 43,000 unfilled nursing vacancies in England in 2019 [9]. Not only are these pressures leading to delayed and poorer quality of care, and a potential rise in mortality [10,11], but they may also be contributing to poor staff mental health and burnout [12,13]. Should a significant number of NHS staff leave the NHS, whether because of Brexit or other reasons, the stresses on the NHS are likely to be intensified.

### Brexit and the hostile environment

Immigrant health workers, like many highly skilled and mobile professionals, are an important “transnational” population [14]. Previous research on migration of health workers has showed that in addition to both objective practical considerations, such as availability of public services like schools, cost of living, as well as work place factors such as staffing levels, teaching and professional support, language and ties to host and origin nation are important factors determining migration [15,16]. Moreover, a recent study highlighted the relationship between Brexit and adverse effects on mood of young doctors connected to political events [17], which demonstrates the importance of socio-political factors acting as push factors in migration decisions.

The Leave campaign ran heavily on anti-immigration rhetoric and the conservative government are proposing strict entry-criteria for new migrants coming to the UK. Thus, it is perhaps unsurprising that this has caused migrants, and in particular, European migrants, to feel unwelcome in the UK, potentially causing many to make plans to leave. Not only might Brexit negatively affect current EU and EEA NHS employees, it may also be linked to a reduction in the number of people coming from Europe to Britain by 70% between 2018–19 [18]. However, it should be noted that according to 2020 guidelines on the points-based system, junior doctors would still qualify for entry to work in the UK.

Brexit may be specifically related to European citizens’ migration in terms of both sense of civic citizenship and subjectively perceived sense of belonging and participation to the UK – what the literature on immigrant transnationalism refers to as transnational subjectivities [14], as well as the objective legal right to live and work in the UK [19]. Even nearly five years after the Brexit referendum, neither the legal status of European citizens in the UK post-Brexit nor the rules for incoming new EU citizens are certain. These legal matters will depend on the future relationship Britain will negotiate with the EU, though the conservative government has made clear that it wishes to break from the Single Market, thus removing the right for Europeans citizens to freely live and work in the UK. Yet, many people, including NHS staff, from outside the EU and EEA continue to come to the UK despite not enjoying the same rights and protections as their EU and EEA counterparts [20]. Such trends suggest that it is the change of status that is impacting the way in which Europeans who reside in the UK perceive their role and belonging in the nation rather than simply the perception of the UK in general.

Evidence suggests that sense of belonging to a nation, the civil component of citizenship, is a significant factor in determining happiness after migration and could consequently influence the chance of return to the home nation [16]. The degree to which belonging and participation to a nation can be measured and considered as an individual marker relative to the decision to migrate is very difficult to quantify. This paper specifically considers national identity as an additional marker for sense of belonging and strength of civil citizenship when examining factors that may influence the departure of European doctors from the UK post-Brexit.

### Rationale for research

Since the result of the referendum there has been concern about the effects of Brexit on NHS staff. Most research has focussed on nurses leaving the NHS as a result of Brexit, most likely due to the initial steep drop of 89% of new EEA nurses entering the registry directly after the referendum [21].There has subsequently been a steady, continuous decline of 13% of the total number of EU and EEA nurses registered to practice in the UK between 2016 and 2018, with a 91% drop in the total number of EU and EEA applicants to join the Nursing and Midwifery register in the same period [22]. A similarly worrying trend is shown by a 2017 British Medical Association survey which found that between 42% (2016) [23] and 45% (2017) of European doctors were considering leaving the UK, while 18% had already made plans to leave [24]. These figures may be misleading as they reflect only doctors who attended medical school and qualified to practice medicine in the EU or EEA, and do not include Europeans that may have trained in England, nor do they reflect those who have legal and or personal connections to Europe who may also be affected by Brexit and may wish to leave the UK. For instance, a British doctor with an EU partner may be negatively impacted by Brexit. It must also be considered that European doctors are unequally geographically distributed, with most clustered in London so their potential absence may be felt disproportionately around the country [6]. Moreover, a survey of Scottish medical students suggested even UK medical students are less likely to practice in the UK because of Brexit, either choosing different career paths or leaving the UK to practice elsewhere [25]. In 2019, partial data released by the Liberal Democrats showed that from less than half the trusts in England, 11,600 European staff including nurses and doctors have left, which may have been precipitated by Brexit. These results imply that Brexit may have been a critical trigger to European staff leaving the NHS. Though many staff have already left, this study aims to conduct a more detailed examination of European doctors’ current intentions to leave the UK as well as examine the factors that may contribute to their potential decisions to migrate.

### Research Question and Aims

This research aims to contribute to the existing body of evidence on how Brexit may impact the UK health care system, specifically by examining how Brexit relates to stated future intentions to leave or remain in the UK among self-identifying European doctors. This study analyses how connectedness to both Europe and the UK as well as demographic factors may relate to self-reported influences of Brexit on migration intentions. Although there have been large-scale surveys conducted about European doctors’ intentions to leave the UK and there has been some qualitative work in the area [26], to our knowledge, this is the first study to use multivariate analysis to examine the relationship between doctors’ decisions about leaving because of Brexit, their British and European identities, and demographics.

## Methods

We fielded a non-probability sample of 59 self-reported doctors who self-identified as EU/EEA/European and working in the UK, which was conducted from 1^st^ March, 2019 to 20^th^ April, 2019. The sample recruitment procedures included advertising the voluntary survey on Facebook groups which serve as forums for medical and political issues for predominantly UK-based doctors working in the NHS as well as through personal contacts, and all EU/EEA/self-identifying European doctors working in the UK were eligible to participate. We rely on these data for analysis. Ethical approval was granted from Queen Mary Ethics Committee (QMREC2257a).

Although doctors from across the UK were eligible to participate in the study, 92% of respondents reported they were working in England. To weight results to the English NHS population [27], we created post-stratification weights that we used to adjust the sample composition to the NHS composition on the following dimensions: sex (woman or man), professional grade level (consultant, core training, foundation, specialty doctor, specialty registrar, or other), and ethnicity (white or other). The use of such weights should increase the applicability of the results to the NHS population and reduce biases owing to the convenience sample [28].

We then ran weighted regression analyses that modeled responses to the following question: “In terms of your future plans, please select which of the following best describes you,” with response options: “1. I am leaving the UK, and Brexit has had an impact on this decision;”, “2. I am leaving the UK, but Brexit has not had any impact on this decision;” “3. I am considering leaving the UK, and Brexit has had an impact on this decision;” “4. I am considering leaving the UK, but Brexit has not had any impact on that decision;” or “5. I am not considering leaving the UK.” We modeled responses to this question in two steps. First, we modeled respondent plans (leaving, considering, not considering) using a multinomial logistic regression. Second, we modeled whether respondents reported Brexit influencing their decision-making, using a binary logistic regression.

In both models, we aimed to descriptively summarize who gives different responses to questions surrounding impact of Brexit and leaving the UK. As such, we included the following variables in the models: self-reported Brexit effects (responses to a question from a low of 0 to a high of 100 how much Brexit impacted the respondents’ professional life, self-described British identity (responses to a question from a low of 0 to a high of 100 how much the respondent identifies as British), self-described European identity (responses to a question from a low of 0 to a high of 100 how much the respondent identifies as European), sex (woman or man), age (continuous by single years), years lived in the UK (less than 5, 5–10, 10–20, or 20+), and professional grade level (consultant, specialty doctor, or other). For both models, we show the table of regression results and the marginal effects associated with focal identity and key demographic variables.

## Results

***Table 1*** shows descriptive statistics for all variables in our analyses. In our sample, a weighted 48% (n = 21) said they were not leaving the UK. The remainder was split between those who said they were considering leaving (weighted 45%; n = 30) and those who were planning to leave (weighted 7%; n = 8). Among those considering leaving, most (n = 7 of 8) reported that Brexit impacted their decision; the same was true among those planning to leave (n = 27 of 30). Overall, participants reported substantial Brexit impacts. For instance, weighted results show they gave an average score of 50 (95% C.I. = [41.2, 59.0]) for Brexit’s impact on professional life. These responses were bimodal and stratified by respondent plans. Those planning to not leave reported substantially lower impacts of Brexit on their professional lives (weighted mean = 30.6, 95% C.I. = [17.5, 43.6]) than those considering leaving (weighted mean = 66.1, 95% C.I. = [55.1, 77.0]) or planning to leave (weighted mean = 79.1, 95% C.I. = [55.5, 102.8]); the latter two groups were statistically indistinguishable.

**Table 1:** Descriptive statistics.


	WEIGHTED PROPORTION/MEAN	UNWEIGHTED SAMPLE SIZE

*Intentions to leave*		

Leaving	7%	8

Considering	45%	30

Remaining	48%	21

*Brexit impact*		

No impact	53%	25

Impact	47%	34

*Sex*		

Female	50%	37

Male	50%	22

*Years lived in UK*		

<5	15%	9

5–10	18%	11

10–20	35%	25

20+	33%	14

*Grade level*		

Consultant	48%	22

Specialty doctor	8%	19

Other	44%	18

*Age*		

Mean [SE]	38.9 [0.9]	59

*Brexit effects scale*		

Mean [SE]	40.1 [4.5]	59

*British identity scale*		

Mean [SE]	35.3 [3.2]	59

*European identity scale*		

Mean [SE]	80.1 [2.5]	59


**Figure 1:**
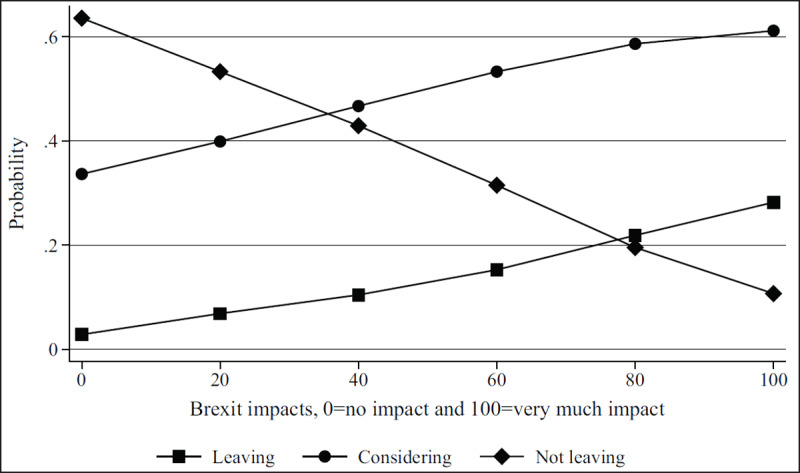
Predicted probability of reporting leaving, considering leaving, and not leaving from multivariable model 1 at different levels of self-reported Brexit impacts on respondent professional lives.

**Figure 2:**
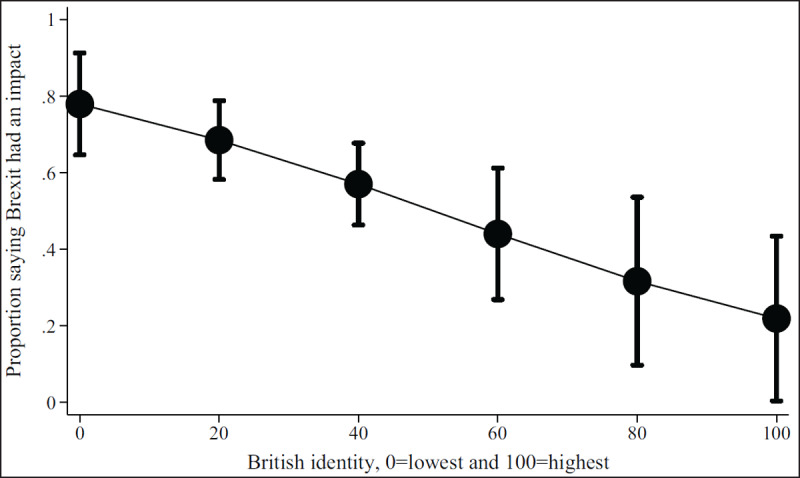
Predicted probability of respondent reporting Brexit had an impact on their decisions about leaving from multivariable model 2 at different levels of self-reported British identity.

**Figure 3:**
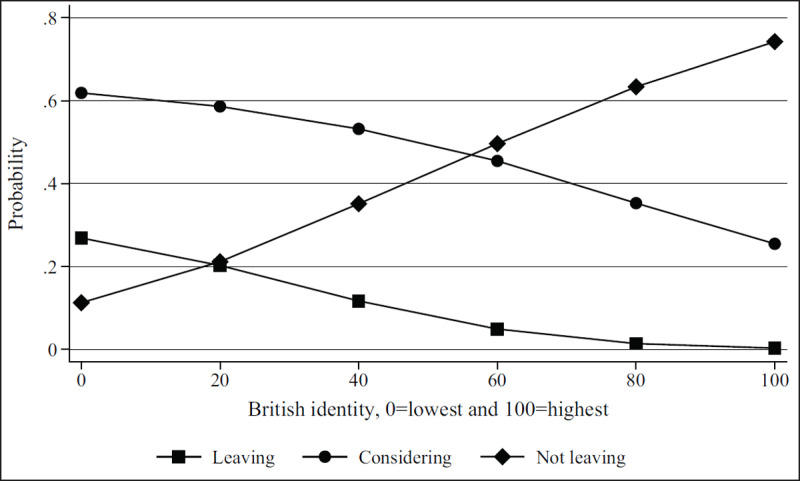
Predicted probability of reporting leaving, considering leaving, and not leaving from multivariable model 1 at different levels of self-reported British identity.

**Figure 4:**
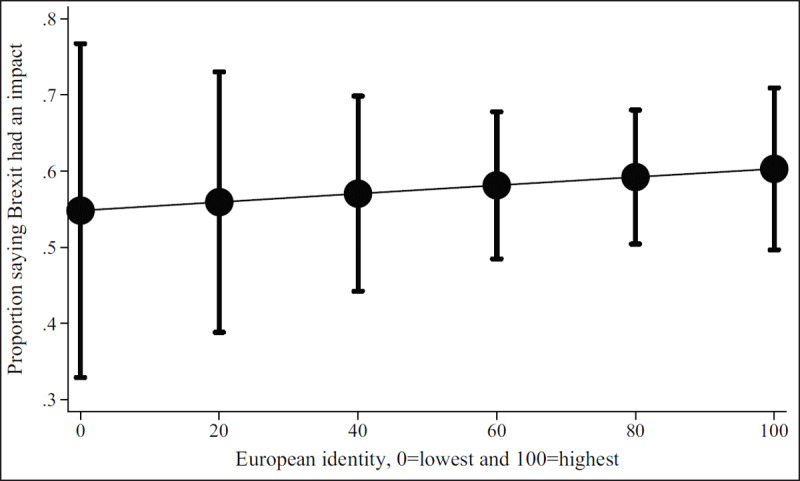
Predicted probability of respondent reporting Brexit had an impact on their decisions about leaving from multivariable model 2 at different levels of self-reported European identity.

**Figure 5:**
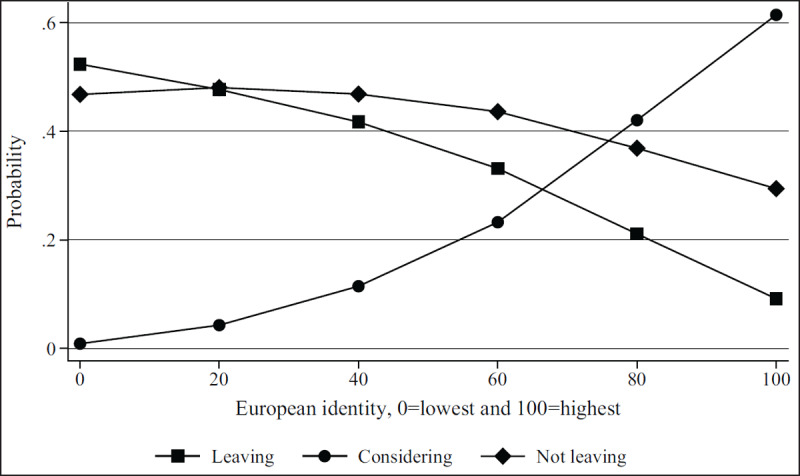
Predicted probability of reporting leaving, considering leaving, and not leaving from multivariable model 1 at different levels of self-reported European identity.

**Figure 6:**
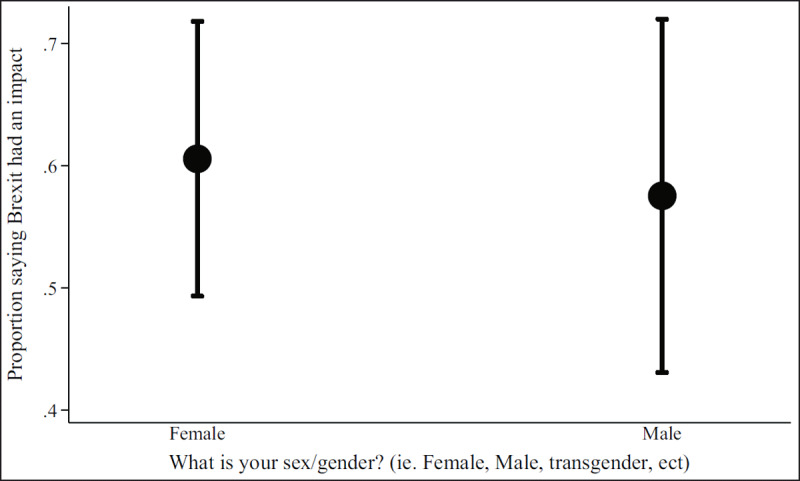
Predicted probability of respondent reporting Brexit had an impact on their decisions about leaving from multivariable model 2 by self-reported gender.

**Figure 7:**
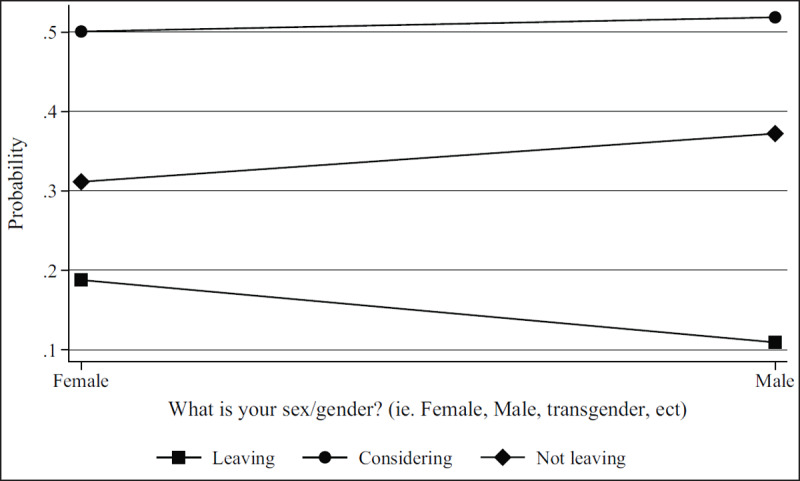
Predicted probability of reporting leaving, considering leaving, and not leaving from multivariable model 1 by self-reported gender.

**Figure 8:**
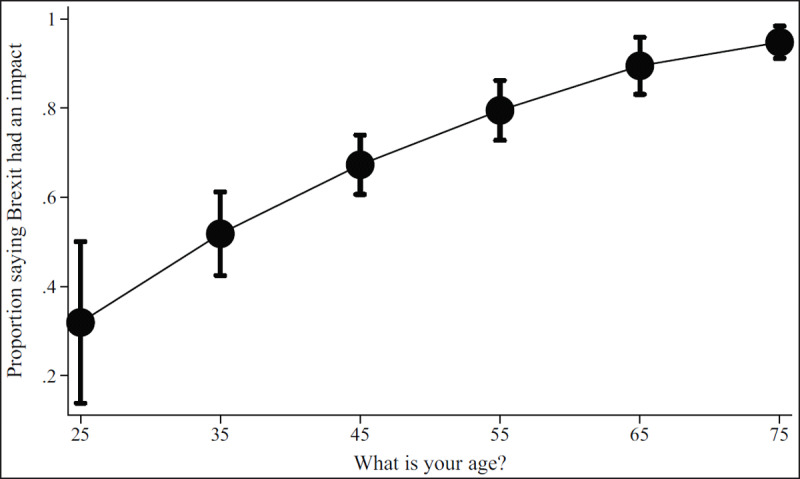
Predicted probability of respondent reporting Brexit had an impact on their decisions about leaving from multivariable model 2 at different ages.

**Figure 9:**
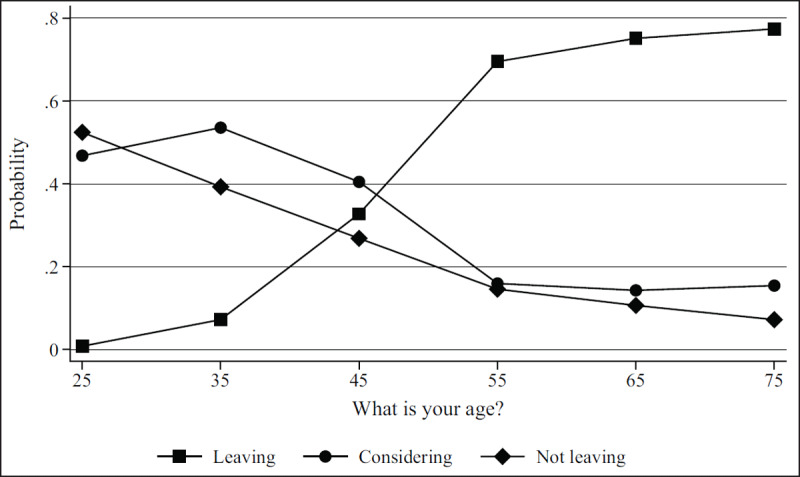
Predicted probability of reporting leaving, considering leaving, and not leaving from multivariable model 1 at different ages.

***Table 2*** shows regression results from the multivariate models. Figures show results by focal identity and key demographic variables. The Brexit impact graph (***Figure 1***) showed that the higher respondents scored that Brexit had impacted their professional lives, the more likely they are to state they will leave or are considering leaving. Results from the British identity graphs (***Figures 2*** and ***3***) showed that those with increased levels of British identity were more likely to state that Brexit does not impact their lives and were not as likely to report planning to leave or considering leaving as those with reduced British identity. While these results were unsurprising, it was unexpected that results from the European identity graphs (***Figures 4*** and ***5***) showed that those with higher levels of European identity felt neither more nor less impacted by Brexit, but were less likely to leave and more sharply more likely to consider leaving compared to those with lower levels of European identity. In terms of demographics, the graphs for gender (***Figures 6*** and ***7***) show that although male and female doctors felt approximately equivalently impacted by Brexit, male doctors were slightly less likely to report that they were leaving than their female counterparts. The age graphs (***Figures 8*** and ***9***) show steadily rising impacts by age and higher probabilities of intending to leave the UK the older one is, with a particularly steep increase and then plateau in the older adult years (above 50).

**Table 2:** Multivariate regression results from both models.


VARIABLES	MODEL 1 (MULTINOMIAL LOGISTIC REGRESSION)			MODEL 2 (LOGISTIC REGRESSION)
	
	LEAVING VS. REMAINING	CONSIDERING VS. REMAINING		IMPACT OF BREXIT

*Sex*				

Female	ref.	ref.		ref.

	ref.	ref.		ref.

Male	–6.7***	–0.7		–0.2

	(1.5)	(0.9)		(1.0)

*Years lived in UK*				

<5	ref.	ref.		ref.

	ref.	ref.		ref.

5–10	13.7***	6.8**		4.6***

	(3.4)	(3.2)		(1.4)

10-20	11.8***	3.7**		3.7**

	(2.4)	(1.6)		(1.4)

20+	–26.0***	0.3		0.6

	(3.5)	(1.2)		(1.2)

*Grade level*				

Consultant	ref.	ref.		ref.

	ref.	ref.		ref.

Specialty doctor	6.0**	8.3***		5.9***

	(2.7)	(2.3)		(1.8)

Other	9.4***	4.1***		4.3**

	(2.1)	(1.4)		(1.7)

*Age*	0.7***	0.2***		0.2***

	(0.2)	(0.1)		(0.1)

*Brexit impacts scale*	0.1***	0.1***		0.1**

	(0.0)	(0.0)		(0.0)

*British identity scale*	–0.2***	–0.1***		–0.1**

	(0.0)	(0.0)		(0.0)

*European identity scale*	–0.1***	0.1***		0.0

	(0.0)	(0.0)		(0.0)

*Constant*	–38.3***	–21.3***		–15.2***

	(8.2)	(5.5)		(4.5)

*Observations*	59	59		59


Notes: standard errors in parentheses; ***p < 0.01, **p < 0.05, *p < 0.10.

## Discussion

This study was the first to quantitatively examine the association between national identity, demographic factors, and the intention of European doctors to leave the UK specifically because of Brexit. Our study shows that interestingly, both higher British and European identity are associated with doctors’ decisions to remain in the UK. It is expected that increased levels of British identity would be associated with remaining in the country, but counterintuitive that higher levels of European identity are not associated with planning to migrate out of the UK. The result may also be related to the surprising finding in the study that those with higher levels of European identity felt less impacted by Brexit than those with lower levels of European identity. This finding could possibly be because those with higher European identity and connectedness to their home country have, even before Brexit, felt separate to the general British population and feel they still have a ‘home’ else-where they can return when they wish to do so. On the other hand, those having a weaker European identity who did not necessarily have a strong British identity, may have been more alienated by the Brexit vote than those who feel strongly European, causing them to consider leaving.

It is intuitive that those European doctors who were more likely to state that Brexit had significantly impacted their professional lives were more likely to leave. Male and older doctors felt more impacted by Brexit than their female and younger counterparts, but male doctors were less likely to voice intentions to leave. This could be because men are advantaged in the NHS in terms of pay and prestige [29] so are less likely to uproot their professional lives. Similarly, the finding that older doctors are more likely to leave may be linked to their willingness to migrate because they are nearing retirement age.

There are several limitations to this study. First, although we apply post-stratification weights that should improve representativeness, the data are based on a non-probability internet sample. Second, the sample is small, which precludes studying nuances in such decision-making by multiple statuses, such as regional differences by sex. In future studies, scholars may seek to sample European doctors in the NHS from existing panel studies or by using other approaches, ideally collecting a large, diverse, and representative sample. Third, we study self-reported intentions to leave and self-assessments of the role that Brexit played in the formation of these intentions. Fourth, these data were collected retrospectively, though close in time to the Brexit event. Parallel research is needed to understand whether there have been, or will in the future be, changes in the rates at which European-identifying doctors leave the NHS and the UK as the Brexit deal is finalized. Although it is valuable to study contemporaneous opinions about factors associated with migration intentions, such intentions may not translate into actual moves.

However, results from this study suggests that a high proportion of doctors (52% of our weighted sample) intend to leave or are considering leaving the UK specifically because of Brexit. In order to retain these individuals in the British medical system, we make several recommendations. First, the NHS should provide financial and legal assistance for doctors related to applying for settled status to reduce the impact felt by Brexit. This has been accomplished in London trusts for nurses [30] as well as in other sectors outside of healthcare [31]. Second, the NHS should seek to make the UK an attractive place to work by increasing investment into retaining and recruiting more staff of all grades, as well as providing financial incentives to European doctors to match what they could gain in other health systems, such as expatriation bonuses. European women, who are more likely to report intentions to leave and on average earn less than their male counterparts, should be specifically targeted. Though it may be legally possible for doctors to stay or in fact enter the UK under new rules, other factors such as national identity will contribute to their decision to stay in or migrate out of the UK. Thus, the focus for healthcare leaders should be to provide the best possible workplace conditions to retain European staff that may otherwise leave the UK. Considering recent immigration changes in the backdrop of COVID-19, we welcome the decision of the government to extend visas free of charge for health workers. However, there will be continued added pressure from the pandemic on the NHS and therefore it is important to continue to support all workers in every way possible for them to be able to stay and work within it.
